# An Explainable MRI-Radiomic Quantum Neural Network to Differentiate Between Large Brain Metastases and High-Grade Glioma Using Quantum Annealing for Feature Selection

**DOI:** 10.1007/s10278-023-00886-x

**Published:** 2023-07-28

**Authors:** Tony Felefly, Camille Roukoz, Georges Fares, Samir Achkar, Sandrine Yazbeck, Philippe Meyer, Manal Kordahi, Fares Azoury, Dolly Nehme Nasr, Elie Nasr, Georges Noël, Ziad Francis

**Affiliations:** 1 Radiation Oncology Department, Hôtel-Dieu de France Hospital, Saint Joseph University, Beirut, Lebanon; 2 ICube Laboratory, University of Strasbourg, Strasbourg, France; 3 Radiation Oncology Department, Hôtel-Dieu de Lévis, Lévis, QC Canada; 4 Physics Department, Saint Joseph University, Beirut, Lebanon; 5 Radiation Oncology Department, Gustave Roussy Cancer Campus, 94805 Villejuif, France; 6 Department of Radiology, University of Maryland School of Medicine, 655 W Baltimore St S, Baltimore, MD 21201 USA; 7 Medical Physics Department, Institut de Cancérologie de Strasbourg (ICANS), 67200 Strasbourg, France; 8 IMAGeS Unit, IRIS Platform, ICube, University of Strasbourg, 67085 Strasbourg Cedex, France; 9 Institut National de Pathologie, Beirut, Lebanon; 10 Radiotherapy Department, Institut de Cancérologie de Strasbourg (ICANS), 67200 Strasbourg, France; 11 Radiobiology Department, IMIS Unit, IRIS Platform, ICube, University of Strasbourg, 67085 Strasbourg Cedex, France; 12 Faculty of Medicine, University of Strasbourg, 67000 Strasbourg, France

**Keywords:** Brain metastasis, Glioma, Machine Learning, Quantum annealing, Variational classifier

## Abstract

Solitary large brain metastases (LBM) and high-grade gliomas (HGG) are sometimes hard to differentiate on MRI. The management differs significantly between these two entities, and non-invasive methods that help differentiate between them are eagerly needed to avoid potentially morbid biopsies and surgical procedures. We explore herein the performance and interpretability of an MRI-radiomics variational quantum neural network (QNN) using a quantum-annealing mutual-information (MI) feature selection approach. We retrospectively included 423 patients with HGG and LBM (> 2 cm) who had a contrast-enhanced T1-weighted (CE-T1) MRI between 2012 and 2019. After exclusion, 72 HGG and 129 LBM were kept. Tumors were manually segmented, and a 5-mm peri-tumoral ring was created. MRI images were pre-processed, and 1813 radiomic features were extracted. A set of best features based on MI was selected. MI and conditional-MI were embedded into a quadratic unconstrained binary optimization (QUBO) formulation that was mapped to an Ising-model and submitted to D’Wave’s quantum annealer to solve for the best combination of 10 features. The 10 selected features were embedded into a 2-qubits QNN using PennyLane library. The model was evaluated for balanced-accuracy (bACC) and area under the receiver operating characteristic curve (ROC-AUC) on the test set. The model performance was benchmarked against two classical models: dense neural networks (DNN) and extreme gradient boosting (XGB). Shapley values were calculated to interpret sample-wise predictions on the test set. The best 10-feature combination included 6 tumor and 4 ring features. For QNN, DNN, and XGB, respectively, training ROC-AUC was 0.86, 0.95, and 0.94; test ROC-AUC was 0.76, 0.75, and 0.79; and test bACC was 0.74, 0.73, and 0.72. The two most influential features were tumor Laplacian-of-Gaussian-GLRLM-Entropy and sphericity. We developed an accurate interpretable QNN model with quantum-informed feature selection to differentiate between LBM and HGG on CE-T1 brain MRI. The model performance is comparable to state-of-the-art classical models.

## Introduction

The most frequent malignant brain tumors in adults are metastases (BM) and high-grade gliomas (HGG) with incidence rates of 7 to 14 and 1.95 per 100,000 population per year, respectively [1, 2]. MRI is the gold standard imaging modality for diagnosing and characterizing BM and HGG. However, differentiating these two entities on MRI is often challenging, owing to shared imaging characteristics such as central necrosis, ring enhancement, and peritumoral edema. Furthermore, both tumor types might present as a solitary mass or multifocal disease, albeit with different frequencies.

The management strategy differs significantly between these two disease categories [3–6]. Although biopsy and/or surgical resection with histopathological and molecular analysis [7] is generally recommended to establish a definite diagnosis and guide personalized treatment, there remains an undeniable need to improve the diagnostic ability of imaging to differentiate between these two disease entities. In rare cases where an invasive procedure could not be undertaken due to medical contraindications or eloquent tumor location, imaging accuracy becomes paramount. Moreover, for patients with a known history of malignancy, avoiding a confirmatory biopsy for brain metastases could prevent unnecessary toxicity, and thus, leveraging confidence in the diagnostic capabilities of MRI imaging is eagerly needed.

MRI techniques that help differentiate between BM and HGG represent an active area of research, and significant improvements over conventional MRI techniques have been made in the last few years using spectroscopy, perfusion, and diffusion-weighted imaging techniques [8–14]. A major drawback of such techniques with respect to conventional MRI is the need for additional special imaging acquisitions that would have a negative impact on the procedural cost, patient convenience, and the time to get a diagnosis. Further, many of these techniques are not widely available.

Radiomics extraction is a promising quantitative method that could glean useful information from imaging data that are not discernable to the human eye [15, 16]. Two types of radiomics exist: “handcrafted radiomics” that are distinct mathematically-defined features generated through a stand-alone process and “deep radiomics” that represent task-oriented features extracted by an auto-encoder or as a part of the deep layers’ data of a convolutional neural network [17]. In many tasks, deep radiomics outperformed engineered features [18–20]. In one BM-glioblastoma classification study by Bae et al. comparing both techniques, deep radiomics yielded slightly better results than their counterparts [21].

Quantum computing is gaining much momentum as advances in quantum device engineering and computational algorithms are surging, and healthcare is a promising arena for these technologies [22]. Quantum machine learning (QML) is an emerging bridging discipline that combines principles from both quantum computing and classical machine learning [23–26]. Few studies have pointed to a potential superiority of QML over classical algorithms under specific circumstances [27–29], and classification tasks with small training datasets might derive some benefit in terms of performance and generalization [30, 31]. However, a clear quantum advantage is yet to be demonstrated. Variational quantum classifiers (VQC), also quoted as quantum neural networks (QNN) due to their analogy with classical neural networks, are hybrid quantum–classical models consisting of three sequential blocks: data encoding or state preparation, trainable parametrized unitary evolution, and measurement of the quantum state. Such algorithms are considered kernel classifiers in that they map classical data to a high-dimensional feature space where a linear separation could be defined by measurement [32]. Although the hope of QML is to achieve an advantage over classical methods through computational speed-ups, such results might not be possible on near-term quantum devices.

In order to enhance model interpretability, dimensionality reduction through feature selection is a pivotal step before training. Mutual information feature selection (MIFS) is a model-agnostic filtering method to select the features that share the maximal amount of information with the output variable and is able to capture non-linear relationships [33]. Conditional mutual information scores have been used to account for feature interaction [34], and such computation is classically intractable. As inspired by the work of Nguyen and colleagues [35], the MIFS problem could be mapped to a quadratic unconstrained binary optimization (QUBO) and solved heuristically using an Ising machine. Quantum annealing is a metaheuristic that consists of evolving an initial multi-qubit system prepared as the ground state of a simple Ising-spin Hamiltonian to a final Hamiltonian whose ground state represents the solution of a desired optimization problem. Quantum annealing has been suggested to match and sometimes outperform classical solvers in combinatorial optimization [36], hypothetically due to quantum tunneling. While quantum annealing is pushing its way towards commercial utilization, its superiority over classical heuristics remains questionable.

Radiomics and deep learning techniques have been widely used for brain tumor classification [21, 37–41]. Deep learning algorithms suffer from the lack of interpretability and are regarded as “black-box” methods, drawing much skepticism around their use in clinical practice. QNNs represent a new family of machine learning algorithms that are interesting to explore on real-world datasets. In this work, we evaluate the performance of a QNN to differentiate between BM and HGG on a real-world conventional MRI dataset, with a particular focus on model interpretability. We use a state-of-the-art method for feature selection, MIFS, taking into consideration feature interaction, and we implement it on a quantum annealer. Moreover, since BM are often smaller than HGG at diagnosis, there exists a significant confounding effect of tumor size that is seldom considered in many published studies [41]. We herein limit our analysis to large BM. Finally, we compare the QNN performance to prove classical machine learning algorithms.

## Materials and Methods

### Patients and Data Collection

This study was conducted at the Hôtel-Dieu de France University Medical Center after obtaining approval from the institutional Ethics board. Medical records from the radiation oncology and radiology departments were retrospectively screened for patients diagnosed with brain metastases and high-grade gliomas (WHO grades 3–4) between 2012 and 2019. A total of 423 patients aged more than 18 years who had a confirmatory pathology report and a pre-treatment gadolinium-enhanced T1-weighted (CE-T1W) brain MRI were included. Brain MRI images were thoroughly reviewed, and patients who had significant motion artifacts, lesions without ring enhancement, or tumors with largest diameter of less than 2 cm were excluded. The final dataset consisted of 129 BM and 72 HGG patients. Imaging performed at our institution as well as imported images from other radiology centers were allowed. Different imaging protocols and 2D or 3D acquisition were permitted as this is more representative of the real-world setting. Since this was a tumor-wise analysis, patients who had multiple brain metastases or multifocal gliomas were accepted for inclusion, yet only one tumor per patient was selected. A 2D largest diameter cutoff of 2 cm was chosen to exclude very small lesions that are more likely to represent metastases rather than gliomas and thus might lead to classification bias.

### Segmentation

Tumors on CE-T1W brain MRI images were manually segmented by a senior radiation oncology resident and a radiation oncologist using Eclipse™ treatment planning system (Varian Medical Systems, Palo Alto, CA, USA). A 5-mm isotropic peritumoral ring extension was created for each tumor volume and was manually edited to carve out parts extending beyond anatomical barriers such as bone and cerebral falx. All segmentations were verified by both physicians for consistency.

### Image Pre-Processing

First, the advanced normalization tools (ANTs) library [42] was used to apply the bias field correction on the MRI images using the N4ITK algorithm [43]. Spatial resampling to a 1 mm × 1 mm × 1 mm voxel size was then performed using ANTs. Automatic brain segmentation was done using two publicly available brain extraction tools, BET from the functional MRI of the brain library (FSL) [44] and the HD-BET [45]. The brain masks were then checked visually, and the best fitting mask was retained for each MRI. The masks were used for Z-score intensity normalization [46] of the resampled MRI images. We used the Z-score method as it resulted in the best performing radiomics-based models when used with a fixed bin number intensity discretization method [47].

### Feature Extraction and Data Pre-Processing

Radiomic extraction was performed using PyRadiomics library version 3.0.1 [48] on Python 3.7.9. Most of the features used in PyRadiomics conform to the imaging biomarker normalization initiative (IBSI) [49, 50]. A fixed bin number of 32 was used for gray-level intensity discretization as recommended by Carré et al. [47]. A total number of 913 tumor features and 900 ring features were extracted for each patient. Seven feature classes were considered: shape features (13 features), first-order statistics (17 features), grey-level co-occurrence matrix (GLCM) (22 features), grey-level run length matrix (GLRLM) (16 features), grey-level size zone matrix (GLSZM) (16 features), grey-level dependence matrix (GLDM) (14 features), and neighborhood grey tone difference matrix (NGTDM) (5 features). Aside from original images, 9 filters were used for first- and second-order features prior to extraction: Laplacian of Gaussian (LoG) (1 filter, sigma value: 1) and wavelet filters (8 filters, with the different combinations of high – H and low – L pass filters in the three dimensions: LLH, LHL, LHH, HLL, HLH, HHL, HHH, and LLL). Shape features were omitted for the ring volume due to irrelevance.

The extracted features were rescaled using Scikit-learn StandardScaler function by subtracting the mean value and scaling to unit variance. The dataset was split into a training set (70%) and a validation set (30%) using a stratified approach. The number of patients in the BM cohort is significantly higher than the HGG dataset. To minimize the effect of class imbalance, a combined over-sampling (synthetic minority oversampling technique (SMOTE)) and under-sampling (RandomUnderSampler) strategy was used. Highly correlated features with a Spearman’s rank correlation coefficient absolute value $|\rho |$ of more than 0.8 were eliminated.

### Feature Selection

Primary feature selection was performed using linear support vector classification (LinearSVC), and then, the best combination of 10 features was determined using MIFS.

Mutual information (MI) between two variables $X$ and $Y$ quantifies the amount of information about one variable derived from observing the other variable. It can be expressed as a function of Shannon entropy (SE) and conditional Shannon entropy (CSE) of these variables as follows:

$$I\left(X;Y\right)=H\left(Y\right)-H(Y|X)$$

where $H\left(Y\right)=-\int p\left(Y\right)\mathrm{log}\;p\left(Y\right)dY$ is the SE of $Y$, a function of its probability distribution $p(Y)$, and $H\left(Y|X\right)=H\left(X,Y\right)-H(X)$ is the CSE of $Y$ conditional of $X$, a function of SE of $X$ and joint SE of both variables.

CMI between two variables $X$ and $Y$ given the previous selection of a variable $Z$ takes into account the interaction between these variables and is given by:

$$I\left(X;Y|Z\right)=H\left(X|Z\right)-H(X|Y,Z)$$

where $H\left(X|Z\right)$ is the CSE of $X$ conditional of $Z$ and $H(X|Y,Z)$ is the CSE of $X$ conditional of $Y$ and $Z$.

Given a set of $n$ features $X=\left\{{X}_{1}, {X}_{2}, {X}_{3}, \dots , {X}_{n}\right\}$, selecting the subset of variables $S\subset X$ that shares the maximum information with a variable $Y$ requires maximizing $I(S;Y)$, which is an NP-hard problem. The method used herein is inspired by the work of Nguyen et al. [35], and a relevant example can be found at [51].

Under the assumption of variable conditional independence, this feature selection problem could be approximated [35] by:

$$\begin{array}{c}\\ \\ \begin{array}{c}arg\;max\\ S\subset X\end{array}\end{array}\left\{\sum_{{X}_{i}\in S}I\left({X}_{i};Y\right)\right.\left.+\sum_{{X}_{i}, {X}_{j}\in S}I\left({X}_{i};Y|{X}_{j}\right)\right\}$$

and could be written in a matrix representation as follows:

$$\begin{aligned}&{\mathrm{arg \;max}} \left\{{x}^{T}\right.\left.Qx\right\}\\&{x\in {\{\mathrm{0,1}\}}^{n}}\end{aligned}$$

where $Q$ is a $n\times n$ matrix with ${Q}_{ii}= I\left({X}_{i};Y\right)$ and ${Q}_{ij}= I\left({X}_{i};Y|{X}_{j}\right)$ for $i\ne j$ and $x$ is a $n\times 1$ vector with $${x}_{i}=\left\{\begin{array}{c}1, {i}^{th} feature\;is\;selected \\ 0, otherwise\end{array}\right.$$  

Evidently, this problem fits to the following QUBO formulation:

$$\mathrm{arg\;min}\left\{-{\sum }_{i}^{n}{Q}_{ii}{x}_{i}\right.\left.-{\sum }_{i<j}^{n}{Q}_{ij}{x}_{i}{x}_{j}\right\}$$

This optimization could be naturally mapped to an Ising model Hamiltonian and thus could be solved by approximating its ground state on an Ising machine. We use for this purpose a quantum annealing approach on D-Wave systems advantage quantum processing unit (QPU).

In order to solve for a specific number of features $k$, a penalty factor is added to the QUBO to penalize solutions with $\left|S\right|\ne k$:

$$P=-\alpha {\left({\sum }_{i=1}^{n}{x}_{i}-k\right)}^{2}$$

where $\alpha$ is a tunable penalty amplitude.

We selected a set of most important features based on MI scores, and then, clique embedding was performed using D-Wave’s minorminer heuristic tool. The MIFS heuristic was solved for all $k$ values, each with 5000 reads. The set of 10 features was selected to build the classification model.

### Quantum Neural Network

We used herein the PennyLane quantum machine learning library [52] to build a QNN, which consists of a parametrized variational circuit that acts as a binary classifier. For this purpose, we used PennyLane’s default_qubit quantum simulator, with a circuit of two qubits using an architecture similar to that described in Farhi and Neven [53] and Schuld et al. [54]. A Python code developed by the PennyLane team to implement a variational classifier could be accessed at [55].

An amplitude encoding method was employed to map the feature vector to the Hilbert space of the 2-qubit system, as shown in Mӧttӧnen et al. [56] and the book by Schuld and Petruccione [26]. For an $n$-qubit circuit, a feature vector of ${2}^{n}$ dimensions is encoded, i.e., four features in the current work. Amplitude encoding entails a sample-wise feature vector normalization to unit norm to satisfy the Born rule, which leads to different normalization factors and significant distortion of the feature dataset. To tackle this issue, three informative features were used and were subsequently padded with a non-zero constant term that would ultimately bear information on the normalization factor.

Principle component analysis (PCA) was used to reduce the dimensionality of the feature dataset from 10 to 3 while retaining the maximal amount of information. Rescaling was performed using MinMaxScaler to ensure positivity of the features, since this would lead to positive amplitudes and thus avoidance of a cascade of Z-axis rotations for the state preparation subroutine [26].

**Fig. 1:**
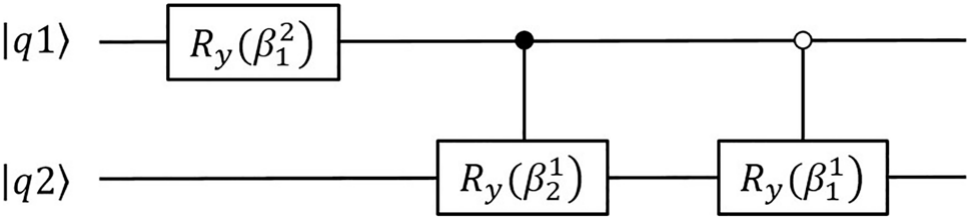
Circuit representation for amplitude encoding in the case of a 2-qubit system. ${R}_{y}\left(\beta \right)$ denotes the Y-axis rotation angle; the white circle indicates a control on qubit basis state 1; the black circle indicates a control on qubit basis state 0; $\left|q1\right.\rangle$ and $\left|q2\right.\rangle$ are qubits 1 and 2 states, respectively

To achieve the amplitude encoding of the normalized feature vectors, a set of controlled Y-axis rotations is performed according to a reversed scheme of the algorithm used by Mӧttӧnen et al. [56]. The corresponding circuit representation in our case is shown in Fig. 1, where $\beta$ denotes the Y-axis rotation angle, and the white and black circles indicate a control on qubit basis state 1 and 0 respectively.

The angles $\beta$ are given by [26]:

$${\beta }_{j}^{s}=2\;{\mathrm{sin}}^{-1}\left(\frac{\sqrt{\sum_{l=1}^{{2}^{s-1}}{\left|{\alpha }_{\left(2j-1\right){2}^{s-1}+l}\right|}^{2}}}{\sqrt{\sum_{l=1}^{{2}^{s}}{\left|{\alpha }_{\left(j-1\right){2}^{s}+l}\right|}^{2}}}\right)$$

where ${\alpha }_{i}$ is the $i$^th^ element of the amplitude vector (i.e., the normalized feature vector).

These controlled Y-axis rotations are further decomposed into basic circuit components including controlled-NOT (CNOT) and rotation around Y-axis (Ry) gates.

**Fig. 2:**
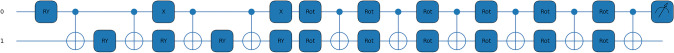
The 2-qubit circuit acting as a machine learning classifier. RY: Y-axis rotation; X: Pauli X gate; Rot: arbitrary qubit rotation. Qubit numbers 1 and 2 are denoted “0” and “1”, respectively

We define a parametrized entangling circuit model composed of 6 layers of a unitary consisting of an arbitrary single qubit rotation (Rot gate) on each qubit followed by a CNOT gate. A Rot gate is parametrized by 3 angle values $\varphi$, $\theta$, and $\omega$ and could be written as a function of Y- and Z-axis rotations as $RZ\left(\omega \right)RY\left(\theta \right)RZ(\varphi )$. This circuit acts on the encoded “ket” vector, and the expectation value of the Pauli Z operator acting on the first qubit is calculated. The quantum circuit (or ansatz) is shown in Fig. 2. If the value is negative, the prediction $p=-1$ is a brain metastasis; otherwise, $p=+1$ for glioma.

The parameters are classically optimized using Adam optimizer, a gradient-descent optimizer with an adaptive learning rate.

### Benchmarking and Model Evaluation

In order to evaluate the performance of the QNN relative to classical machine learning models on our 10-features dataset, we benchmarked our results against two well-established classical models: extreme gradient boosting (XGBoost) and dense neural network (DNN).

For XGBoost, we used Bayesian optimization to tune 9 hyper-parameter tuning to get the best area under receiver operating characteristic curve (ROC-AUC). The search range for the hyper-parameters was $1\times {10}^{-5}$ to $1\times {10}^{-1}$ for the learning rate, 300 to 1000 for the number of trees, 1 to 4 for the minimum sum of instance weight needed in a child, 3 to 5 for maximum tree depth, 0.2 to 0.5 for the subsample ratio of the training instance, 0.2 to 0.5 for the subsample ratio of columns when constructing each tree, 0 to 0.1 for the minimum loss reduction, and 0 to 75 for L1 and L2 regularization terms on weights. Five-fold cross-validation was done on the training set to reduce overfitting.

The DNN used Keras’ Adam optimizer, and included three hidden layers, with the output layer using a sigmoid activation function. We used a dropout layer with a rate of 30% after the input and each hidden layer to minimize overfitting. Early stopping with a patience of 10 was utilized during training.

Balanced accuracy (bACC) and ROC-AUC were used for model comparison. Precision, recall, and F1-scores were also reported.

### QNN Model Interpretation

Shapley value [57, 58], a coalitional game-theory solution concept to determine a player’s contribution to an overall cooperative gain, was used herein to compute sample-wise feature attribution to an overall prediction, as implemented by Lundberg and colleagues [59]. We used the model-agnostic KernelExplainer to estimate Shapley values for the purpose of the current study. The mean absolute Shapley values for all features were calculated for impact ranking. Class-wise mean Shapley values and mean feature values were also calculated, and the same was done for true and false predictions.

## Results

### Patient Characteristics

The BM cohort included 56 patients (43.4%) with primary lung cancer, 40 patients (31%) with breast cancer, and 33 patients (25.6%) with other histologies. Median tumor 3D-largest diameter was 34 mm (IQR: 26–42.6 mm) for BM and 47.9 mm (IQR: 38.4–61.1 mm) for HGG. 3D MRI acquisition was performed in 91 patients with BM (70.5%) and 43 patients with HGG (59.7%).

### Feature Selection

**Fig. 3:**
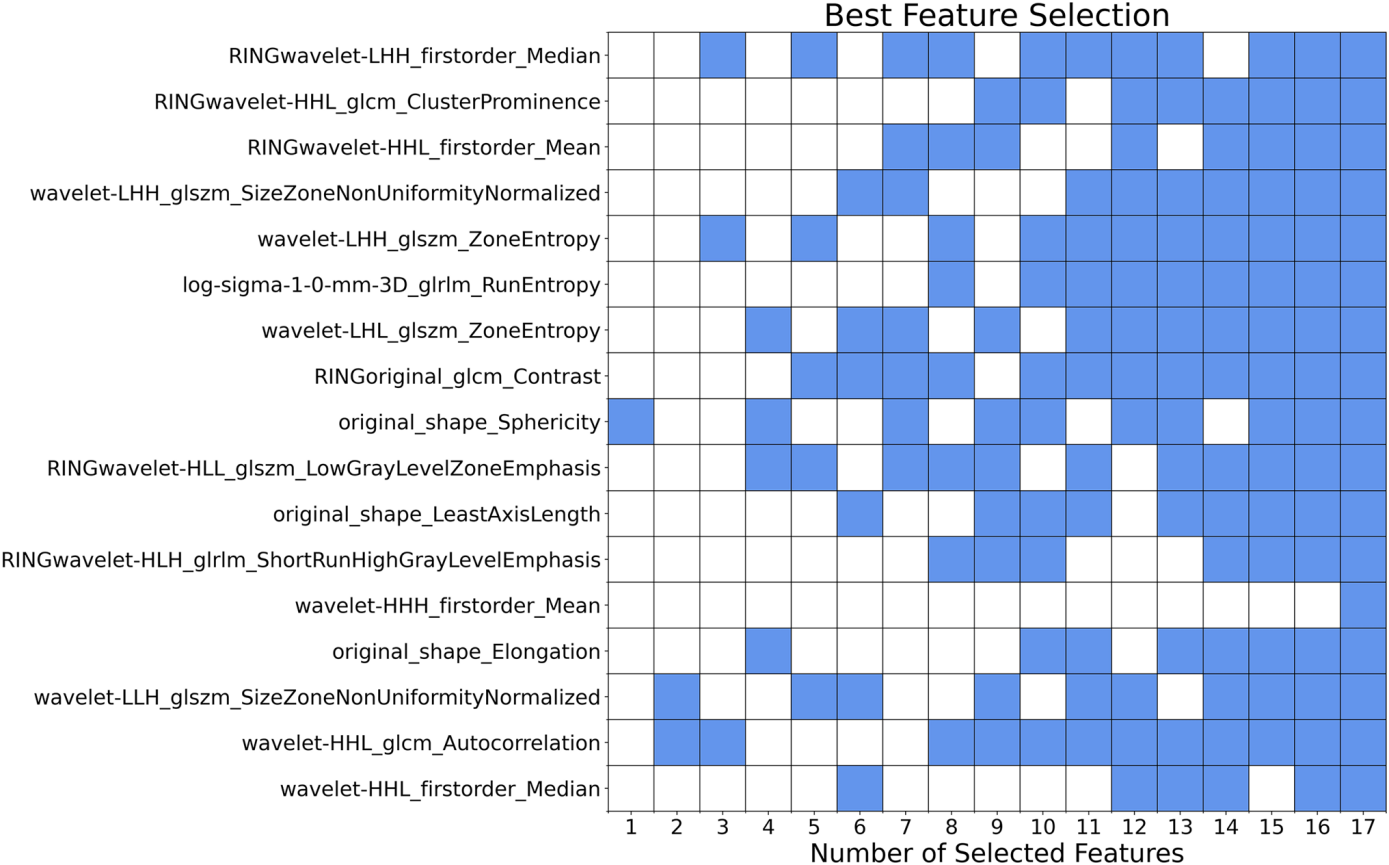
The results of the MIFS heuristic for all $k$ values between 1 and 17, showing the best combination of $k$ features. Blue squares correspond to selected features

After eliminating highly correlated features, a set of 209 features was kept. Using LinearSVC, 54 important features were classically selected. The MI scores for these remaining features were calculated, and 17 most important features were retained. The MIFS heuristic was solved on D-Wave’s quantum annealer for $k$ between 1 and 17 (Fig. 3). The best combination of 10 features was determined and was used to build the machine learning classifiers.

### QNN Model Performance and Benchmarking

**Fig. 4:**
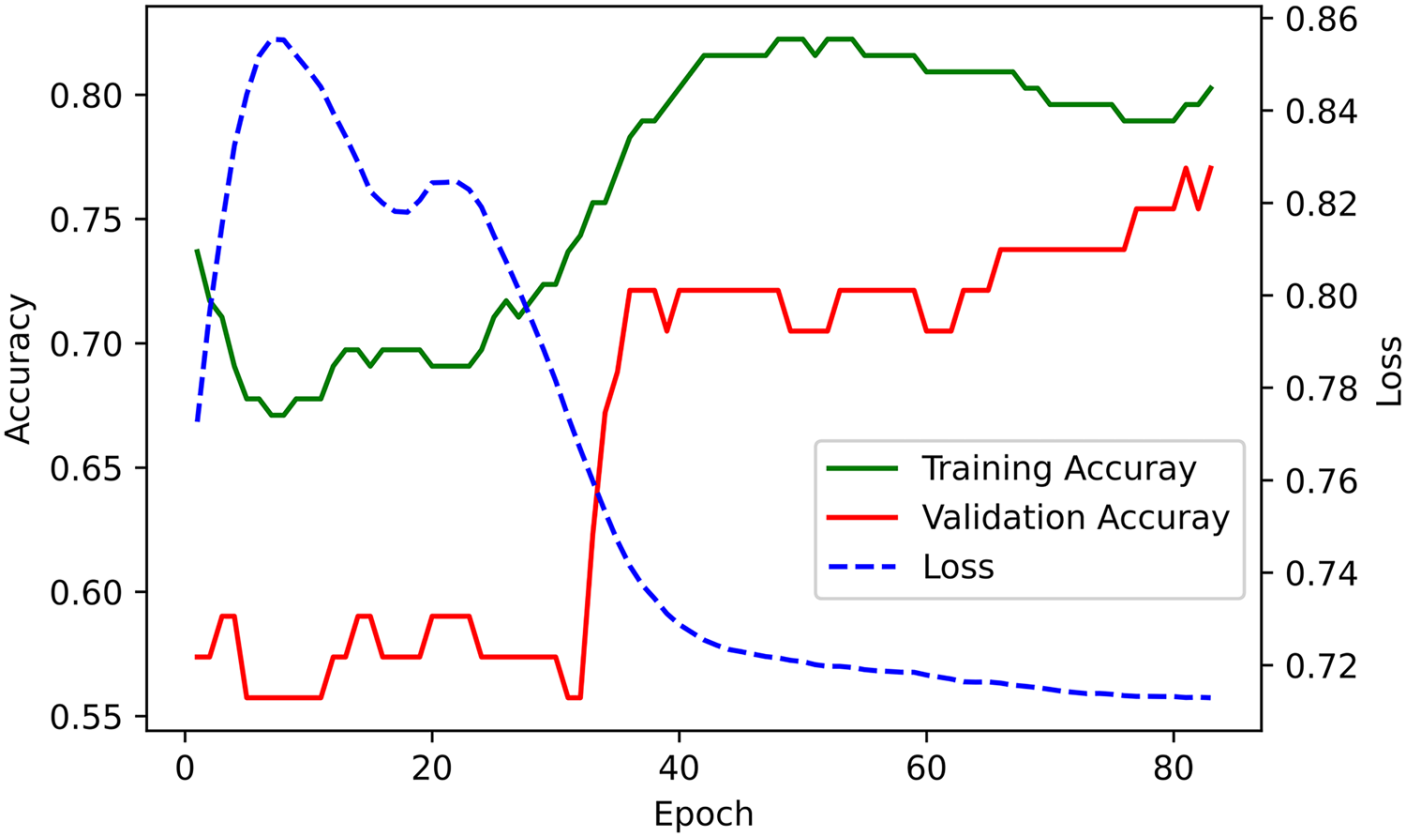
Learning curves for training and test accuracy and training loss for the quantum neural network model

The variational quantum classifier training and validation accuracy learning curves, as well as the training loss curve, are shown in Fig. 4. Model training accuracy was 0.8, and validation bACC was 0.74. ROC-AUC was 0.86 on the training set and 0.76 on the validation set.

Respective bACC on the test set for XGBoost and DNN were 0.72 and 0.73. Training and test ROC-AUC were 0.94 and 0.79 and 0.95 and 0.75 for XGBoost and DNN, respectively. These metrics along with the precision, recall, and F1-scores for all models are summarized in Table 1. The difference in accuracy between training and validation sets are 6%, 17%, and 15% for QNN, XGBoost, and DNN, respectively, and the respective differences in ROC-AUC are 10%, 15%, and 20%.

**Table 1:** Models’ performance metrics

		QNN	XGBoost	DNN
Training ACC		0.8	0.89	0.88
Test bACC		0.74	0.72	0.73
Training ROC-AUC		0.86	0.94	0.95
Test ROC-AUC		0.76	0.79	0.75
Test precision		0.77	0.75	0.77
	BM	0.80	0.79	0.79
	HGG	0.7	0.68	0.72
Test recall		0.77	0.75	0.77
	BM	0.85	0.85	0.87
	HGG	0.64	0.59	0.59
Test F1-score		0.77	0.75	0.76
	BM	0.83	0.81	0.83
	HGG	0.67	0.63	0.65

*QNN quantum neural network, XGBoost* extreme gradient boosting, *DNN* dense neural network, *ACC* accuracy, *bACC* balanced accuracy, *ROC-AUC* area under receiver operating characteristic curve, *BM* brain metastases, *HGG* high-grade glioma

### QNN Model Interpretability

**Fig. 5:**
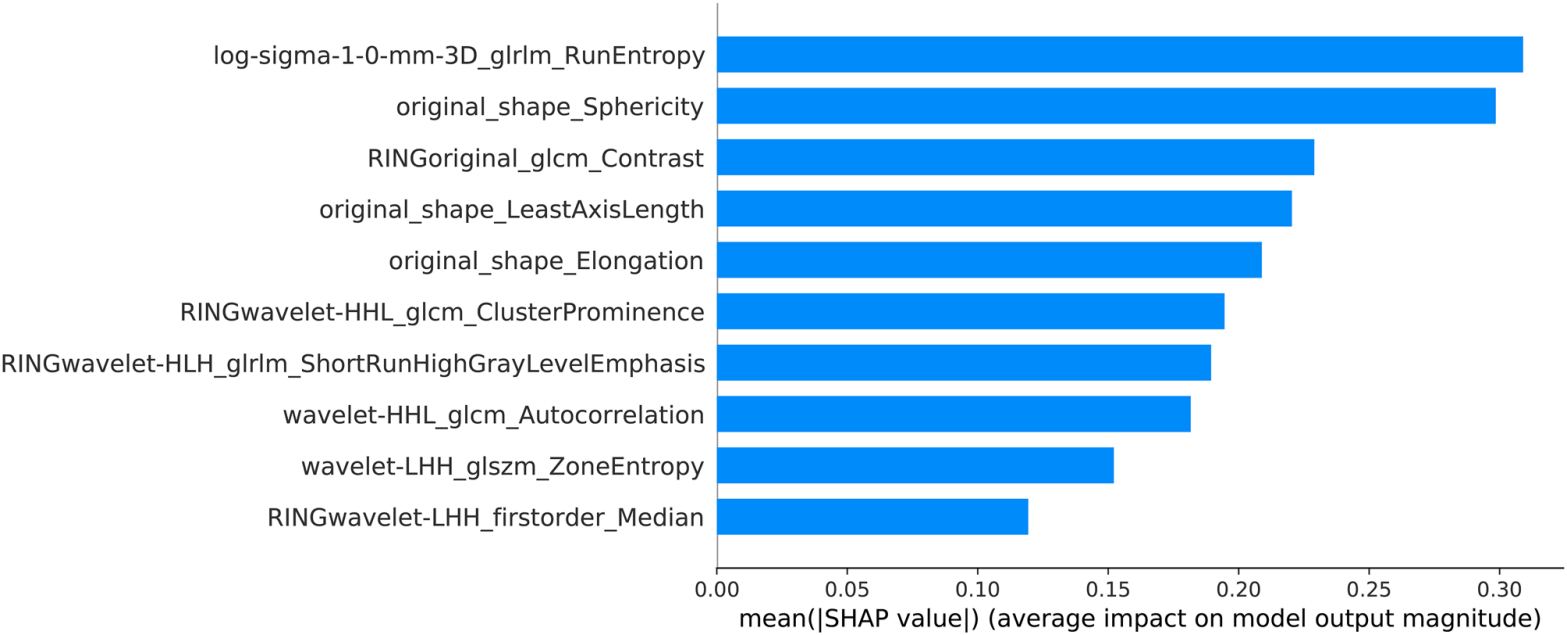
Mean absolute Shapley value for each feature, ranked by order of importance

Instance-wise Shapley values were calculated, and each feature’s contribution to the overall model prediction was determined. Figure 5 shows the mean absolute values for each feature, ranked by order of importance. Tumor LoG-3D-GLRLM-Run-Entropy and sphericity were the most important features. Ring GLCM-Contrast ranked third, and was the most important ring feature.

**Fig. 6:**
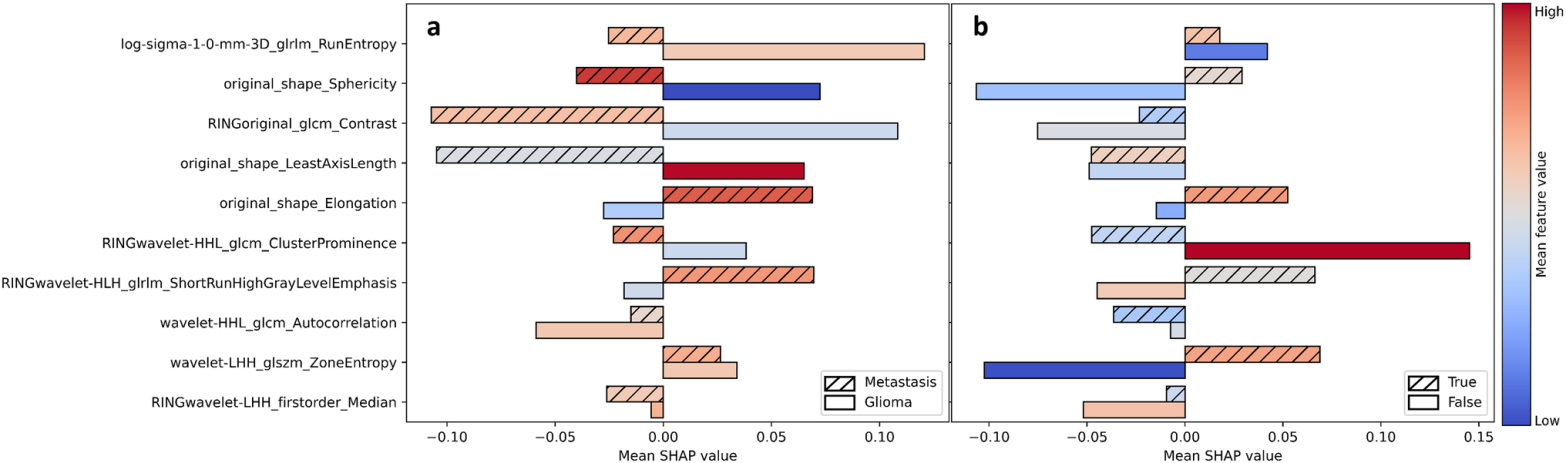
Mean Shapley values and feature values for the different features according to the predicted class (metastasis or glioma) (**a**) and the prediction accuracy (true or false) (**b**). Colors represent the mean feature values according to the color scale bar

Figure 6a shows the mean Shapley value for each feature according to the predicted class, and colors represent the mean feature value according to the color scale bar. A positive Shapley value shifts the prediction output towards HGG from the baseline value, i.e., the mean Shapley value for all predictions. For instance, low tumor sphericity is associated with a positive Shapley value for HGG predictions, whereas for BM, a high sphericity value leads to an average negative Shapley value. Further, a high tumor LoG-3D-GLRLM-Run-Entropy that indicates increased texture heterogeneity contributes to negative Shapley values and shifts the prediction towards BM. A lower average GLRLM-Run-Entropy value is associated with a high positive mean Shapley value shifting the outcome significantly towards HGG. On another hand, high values of the two most important ring features, GLCM-Contrast (correlating with disparity in intensity values among neighboring voxels) and Wavelet-HHL-GLCM-Cluster-Prominence (implying more asymmetry about the mean voxel value), are associated with negative Shapley values pushing the prediction towards BM, and the opposite is true for HGG.

Figure 6b highlights differences in Shapley value and feature values between true and false predictions on the test set. The largest mean Shapley value magnitudes for the false predictions were observed for low tumor sphericity combined with a negative Shapley value, high ring Wavelet-HHL-GLCM-Cluster-Prominence combined with a positive Shapley value, and low tumor Wavelet-LHH-GLSZM-Zone-Entropy combined with a negative Shapley value.

## Discussion

The integration of machine learning models in conjunction with other clinical instruments holds the potential to significantly enhance the accuracy of diagnosing and planning treatment for brain tumors, specifically LBM and HGG, using MRI scans. By leveraging the power of quantum computing, a QNN model can uncover subtle patterns and relationships within radiomic data, leading to more precise and reliable classification of brain tumors. This enhanced accuracy can aid healthcare professionals in making more informed decisions regarding treatment strategies, such as surgical intervention, radiation therapy, or targeted drug therapies. Developing an accurate QNN model could serve as a valuable tool for non-invasive and automated analysis of medical imaging data, reducing subjectivity and interobserver variability in tumor classification.

In this work, we developed an interpretable radiomic classifier to differentiate between large BM and HGG on CE-T1W brain MRI. We used to this end a QNN model, and we showed that its performance on our dataset is comparable to two state-of-the-art classical algorithms: XGBoost and DNN. We employed a MIFS method and solved the resulting combinatorial optimization on D-Wave’s quantum annealer. To the extent of our knowledge, this is the first study to use quantum algorithms for brain tumor classification on a real-world dataset, and our results shed light on the potential value of these algorithms and the need for further exploration and development.

Since the role of feature selection is cardinal for reducing model complexity and leveraging interpretability, we applied a pipeline of two feature selection methods as this strategy was shown to improve the selection results [60, 61]. We used a MIFS method to get the best 10 features, since this method captures non-linear relationships and accounts for interaction effects between features. The resulting QUBO, a computationally expensive optimization, was solved using a quantum annealing metaheuristic as it was shown to match or outperform other classical methods [36]. Nonetheless, the limited number of qubits and connectivity in quantum annealers can restrict the scale and complexity of implementable circuits, and this remains a considerable limitation in the current era.

While the QNN model developed herein showed similar performance on the test set as for XGBoost and DNN, the difference between training and validation metrics was smaller for QNN than for the classical algorithms despite using overfitting techniques for the latter, hinting to a better generalization and lower overfitting for the QNN. This is in line with the work of Caro et al. that showed a favorable generalization error for quantum machine learning algorithms [31], and it would be worthwhile testing this further in future studies.

Differentiating between BM and HGG has been the subject of many previously published studies [41], including handcrafted radiomics-based machine learning techniques as well as deep learning models, with reported accuracy results ranging from 64 to 98%. Comparing results indirectly from separate studies is trivial, and such heterogeneity in classification performance could stem from many factors including the nature and quality of the data, the methods used, and the quality of reporting. In a systematic review by Jekel et al. [41], less than half of the TRIPOD items reflecting critical points in model development were reported on average. Moreover, most of the published papers do not report the tumor size, which could introduce significant classification bias due to the fact that BM are more likely to be diagnosed at an earlier growth stage than HGG owing to the close surveillance pattern of cancer patients.

de Causans and colleagues [62] developed a tumor-radiomics machine learning algorithm based on CE-T1W MRI to classify glioblastoma and solitary BM larger than 2cm, yielding a balanced accuracy of 0.8 and a ROC-AUC of 0.85 on the test set. Their results were slightly better than ours; however, it should be noted that imaging was done on the same 3-Tesla MRI machine using the same protocol, by contrast to our heterogeneous dataset including images from different institutions using different machines and acquisition parameters. Although heterogeneity of training data might affect the performance of a model, its ability to generalize to unseen data with different acquisition protocols might be better. Nonetheless, the ultimate test of generalization is through external validation. Heterogeneity has been shown to compromise the robustness of texture features, yet most of the selected texture features in this work were shown to have good repeatability for T1-weighted MRI with various MRI scanners and scanning parameters, particularly after pre-processing [63]. In this study, we adhered to the image pre-processing pipeline suggested by Carré et al. [47] to maximize radiomic feature stability. Moreover, 3 out of 10 features were shape-based and thus are very robust with respect to imaging techniques.

In this work, we focused on the model interpretability. Sphericity ranked second in terms of feature attribution to an overall prediction. This is consistent with the results of de Causans et al. [62] and Priya et al. [64] where tumor sphericity was the most important discriminating feature. From a clinical perspective, being able to see how each feature is influencing the decision process for a certain prediction would give more confidence in using the algorithm in practice. For instance, knowing that BM are more spherical on average than HGG, if an algorithm uses low sphericity of a tumor to significantly bias a prediction towards a BM prediction, the probability that this prediction is false increases. Our results support this rationality, and as we can see in Fig. 6b for sphericity, false predictions had high mean absolute Shapley values attributing low sphericity to BM prediction. We acknowledge that some features are more complex to understand, and significant uncertainty remains as to extrapolating average values to individual instances.

Our analysis had some limitations. Despite being one of the largest studies to report on this subject, the sample size remains relatively small. Additionally, in this study, external validation was not conducted to provide a better evaluation of generalizability, although the heterogeneous nature of our dataset could potentially be advantageous in this aspect. This highlights the difficulty in gathering sufficient data for imaging-based machine learning studies in neuro-oncology and stresses on the need for data-sharing initiatives to improve model development and validation and push this field further towards clinical integration. Furthermore, we limited our model to CE-T1W MRI sequence. Adding more sequences might lead to improved prediction performance, although it might increase model complexity.

We used PennyLane’s quantum simulator for the purpose of this study since our scope is to test the algorithm rather than the quantum device. Nevertheless, this algorithm could be easily implemented on a near-term quantum computer with reasonable error. The model architecture is scalable and could include a larger number of features; however, as the number of required qubits increases, the circuit becomes exponentially expensive to simulate classically. The rapid development in quantum device engineering and quantum error-correction algorithms is likely to allow practical implementation of such algorithms in the near future. However, we acknowledge that quantum technology is still in its early stages, and further developments are needed to fully exploit the advantages of quantum computing over its classical counterpart. Efforts are eagerly needed to explore and establish the utility of quantum machine learning algorithms in the field of medicine, more particularly in oncology.

## Conclusion

In conclusion, this work led to the creation of a cutting-edge CE-T1W MRI-based radiomic quantum neural network classifier, employing quantum-informed mutual information feature selection. We were able to demonstrate that our developed model effectively discerns between large brain metastases and high-grade gliomas with remarkable accuracy. Notably, our model’s performance was found to be on par with two prevailing state-of-the-art algorithms, namely, XGBoost and dense neural network, while seemingly exhibiting less susceptibility to overfitting issues. This algorithm warrants further external validation. We have also provided a game-theory approach for model interpretability using Shapley value, and our results were in line with published data. Further development of such algorithms using quantum technology has significant implications for the field of medical imaging, paving the way for enhanced diagnostic capabilities and improved patient care in the realm of brain tumor classification.

## Data Availability

The data that support the findings of this study are available upon reasonable request from the corresponding author. The data are not publicly available due to privacy and ethical restrictions.
